# Westminster Medical Society, 16th February, 1833

**Published:** 1833-03

**Authors:** 


### REPORTS
### FROM HOSPITALS AND MEDICAL SOCIETIES.

### WESTMINSTER MEDICAL SOCIETY, 16th February, 1833.

*Effects of large Doses of Quinine.*

Drs. James Johnson and Granville having, at a former meeting, mentioned the peculiar effects witnessed in the case of a medical friend, Mr. Scott, who had taken what might be called excessive doses of quinine; Mr. S. being present this evening, he, at the request of the president, detailed his own case at length; the chief features of which are as follows.

From early life, Mr. S. had been the victim of dyspepsia; but, although his stomach was a perpetual source of uneasiness, his bowels had never in any way troubled him, until, in the year 1829, he was suddenly seized with general and violent pains in the abdomen, of so excruciating a character as to defy description. After the use of opiate enemata, the paroxysms subsided; but recurred regularly for several successive nights, and at length even opium failed to afford relief. Turpentine clysters were next resorted to, which acted like a charm; the pains subsided, and no relapse occurred for an entire year: at the end of this period they returned, with, if possible, increased violence, and recurred several times a-day; and, the intermissions getting shorter, they at length were almost constant. Now even the turpentine enemata failed to assuage the agony, and a variety of treatment was followed, under the advice of Dr. Johnson, with ultimate benefit.

The attacks, however, though lessened in duration and in frequency, were not subdued, and Mr. S. subsequently consulted another physician, who recommended quinine, looking on the disease as intermittent neuralgia, but telling him, at the same time, that he must not expect relief excepting from *very large* doses. He was desired to commence with two grains three times a day, and to increase each dose a grain every other day, until they arrived at twenty grains; i. e. a drachm a day. Liberal diet was at the same time allowed.

Until the doses were increased to fourteen or sixteen grains, he did not experience any peculiar effects; but then he began to feel heat of skin, dryness of mouth and fauces, and obstinate costiveness. He likewise, at the same time, lost the power of naming substantives; was obliged for a long while to consider what different familiar things were called; and as to casting up a line of six or eight figures correctly, it was utterly impossible, he could never make the amount twice alike. His perceptions of quantity were greatly impaired, so that, in prescriptions, he wrote ounces instead of drachms, ordered drachms instead of grains, directed draughts to be put in gallipots, and prescribed fluids to be made into pills.

Notwithstanding these symptoms, he still persevered with the sulphate of quinine, until he took a scruple four times a-day. He was, however, unable to continue these excessive doses long; for the untoward symptoms above detailed increased, so that he was often unable to stand, and several times fell suddenly when in the streets. He, therefore, discontinued the quinine; but, after a short interval, he recommenced its use, at the suggestion of the same physician who at first prescribed it. Now he found that he could not exceed eight-grain doses; for, when he arrived at that quantity, the same effects ensued as from the former scruple doses, and he consequently left it off altogether.

Mr. S. added, that he was unable to take any, even the mildest, purgative, without provoking the return of one of these excruciating paroxysms, and that he regulated the alvine excretions by warm water and enemata.

Dr. Johnson, who was present, corroborated Mr. Scott's statement, and observed, that he had watched the case with great attention. He at first believed it to be intermittent neuralgia, but subsequent observations, and the most patient reflections, had convinced him that the abdominal pains were referable to some mechanical cause, which it was his opinion would never be removed. He thought that many slight inflammatory attacks had probably caused adhesions to be formed between the enteric convolutions; and hence, when the peristaltic action was increased by purgatives, the pains recurred; and that the paroxysms were owing to some portion of the intestines becoming involved in these entanglements and adhesions. He thought it better to state this even in the presence of the patient, as less could be expected from the influence of medicine than from extreme ease, in which he considered Mr. S. somewhat deficient, more so than he thought he should be, both in diet and exposure, if similarly affected. He added, that he had himself taken large doses of quinine, and had experienced analogous symptoms to some of those mentioned by Mr. Scott; but that his sensations were all of the most pleasurable kind.

